# Supplementary material for: Ginkgo biloba extract inhibits hippocampal neuronal injury caused by mitochondrial oxidative stress in a rat model of Alzheimer’s disease
Source: PLoS One. 2024 Aug 6;19(8):e0307735. doi: 10.1371/journal.pone.0307735 (PMC11302898; doi:10.1371/journal.pone.0307735)
Supplement: Attachment — Submitted filename: Minor Revision.pdf [file pone.0307735.s002.pdf]

Minor Revision

I:

Wrong	Right
ginkgo biloba	*Ginkgo biloba*
ad libitum	*ad libitum*
in vitro<br>in vivo	*in vitro*<br>*in vivo*

II: Reference for GBE, $A\beta_{25-35}$, Vitamin E, and CMC doses.

III: Writing the references does not match the Plos one journal method.
