# Supplementary material for: Ginkgo biloba extract inhibits hippocampal neuronal injury caused by mitochondrial oxidative stress in a rat model of Alzheimer’s disease
Source: PLoS One. 2024 Aug 6;19(8):e0307735. doi: 10.1371/journal.pone.0307735 (PMC11302898; doi:10.1371/journal.pone.0307735)
Supplement: Attachment — Submitted filename: Response to Reviewers.docx [file pone.0307735.s003.docx]

**Journal: PLOS NOE**

**Title:** *Ginkgo biloba* extract inhibits hippocampal neuronal injury caused by mitochondrial oxidative stress in Alzheimer's disease rat

**PONE-D-23-22230**

**Response to Reviewers**

**Dear Dr. Olasehinde and reviewers,**

First I would like to thank you for giving us the opportunity to submit a revised draft of the manuscript “*Ginkgo biloba* extract inhibits hippocampal neuronal injury caused by mitochondrial oxidative stress in Alzheimer's disease rat” for publication in the Journal of PLOS ONE. We sincerely appreciate the time and effort that you and three reviewers dedicated to provide the valuable comments on our manuscript. All the insightful comments were considered carefully and the manuscript was revised with tracked changes as necessary based on the suggestions made by reviewers. Attached please find the point-by-point response to the reviewers’ comments and concerns. Should you have any questions or concerns, please contact me.

Sincerely,

Chenyi Xia

**Editor’s Comments to the Authors:**

**Abstract**

The concentration of the *Ginkgo biloba* extract 50 was not specified.

**Author response:** The concentration of the *Ginkgo biloba* extract 50 solution was 7.5 g/L and 15 g/L two concentrations.

Administration of GBE50 can reverse the activity of these three enzymes and reduce the level of H_2_O_2_ – this is not clear. The study has been done already, why did authors indicate it can reverse.

**Author response:** The word “reverse” is not correct and has been replaced with “regulate”. Administration of GBE50 can regulate the activity of three enzymes.

**Methods**

Section 2.2 animals - Male SD rats (220 ± 10 g) – what kind of animals were used? SD is not defined.

**Author response:** Section 2.2 animals, the animals were Male Sprague-Dawley rats (220 ± 10 g).

Line 94 – what is concentration of Aβ_25-35_ used?

How was Aβ_25-35_ prepared and aggregated? For how long was it prepared before administration. Was it a once off administration or continuous?

**Author response:** Line 94. The concentration of Aβ_25-35_ was 5 μg/μl and it was incubated for aggregation at 37℃ for 4 days, which were introduced at Line 76 -78 of the manuscript. After that, it was injected into the hippocampus CA1 region at once. The methods of preparation and aggregation for Aβ_25-35_ were obtained from previous studies [1, 2].

[1] Alkam T, Nitta A, Mizoguchi H, Itoh A, Nabeshima T. A natural scavenger of peroxynitrites, rosmarinic acid, protects against impairment of memory induced by Abeta(25-35). Behav Brain Res. 2007;180(2):139-45.
[2] Klementiev B, Novikova T, Novitskaya V, Walmod PS, Dmytriyeva O, Pakkenberg B, et al. A neural cell adhesion molecule-derived peptide reduces neuropathological signs and cognitive impairment induced by Abeta25-35. Neuroscience. 2007;145(1):209-24.

How did the authors confirm that the rats had Alzheimer’s disease before administering the drug? Was a confirmatory test done on all the rats before GBK was administered?

Alzheimer’s disease is a progressive disease and may be developed overtime especially. Why did authors choose to administer GBK a day after Aβ_25-35_ injection?

**Author response:** The major component of senile plaques in the brain of AD is Aβ _1–40_ and Aβ_1–42_. The core toxic fragment of full length Aβ_1–40_ is Aβ_25–35_ [1]. Some early research had used as AD-like dementia model by the intracerebroventricular and hippocampal injection of Aβ_25–35_, and the animals shew the cognitive impairment and oxidative damage on few days after the injection of Aβ_25–35_ [1-3]. In this study, we referred to these researches to establish this AD-like dementia model and evaluated the antioxidant effects of GBE50 administering after injection, this drug administration used as a precautional or therapeutic measures.

[1] Alkam T, Nitta A, Mizoguchi H, Itoh A, Nabeshima T. A natural scavenger of peroxynitrites, rosmarinic acid, protects against impairment of memory induced by Abeta(25-35). Behav Brain Res. 2007;180(2):139-45.
[2] Klementiev B, Novikova T, Novitskaya V, Walmod PS, Dmytriyeva O, Pakkenberg B, et al. A neural cell adhesion molecule-derived peptide reduces neuropathological signs and cognitive impairment induced by Abeta25-35. Neuroscience. 2007;145(1):209-24.
[3] Shen YX, Xu SY, Wei W, Sun XX, Liu LH, Yang J, Dong C. The protective effects of melatonin from oxidative damage induced by amyloid beta-peptide 25–35 in middle-aged rats. J. Pineal Res. 2002; 32:85–89.

What is the justification for the dose (n at dose of 75 or 150 mg/kg) and 15 days treatment.

**Author response:** According to some studies about GBE50 purchased from the same company, the rats were treated by different dose of GBE50 (20, 40, or 60 mg/kg body weight) by oral gavage for 15days in ischemia reperfusion [1], and the mice were given gavage of 25 mg/kg, 50 mg/kg and 100 mg/kg GBE50 every day from 3 to 15 weeks in high-fat diet [2]. We chose two doses of GBE50 (75 mg/kg and 150 mg/kg) that used usually in our research team and treated rats every day by oral gavage for 15 days after hippocampal injection of Aβ_25–35_ in our study.

[1] Lu SP, Guo X, Zhao PT. Effect of Ginkgo biloba extract 50 on immunity and antioxidant enzyme activities in ischemia reperfusion rats. Molecules. 2011;16(11):9194-206.
[2] Li L, Yang L, Yang F, Zhao XL, Xue SJ, Gong FH. Ginkgo biloba Extract 50 (GBE50) Ameliorates Insulin Resistance, Hepatic Steatosis and Liver Injury in High Fat Diet-Fed Mice. J Inflamm Res. 2021;14:1959-1971.

Line 103 – authors indicated Behaviour Tests but only one test (morris water maze) was done. Why did authors conduct only behavioural test. This cannot reveal a true picture behavioural function of the animal.

**Author response:** Great advices for our study doing better. The previous data had demonstrated that the injection of Aβ_25–35_ into hippocampus of rats induced marked memory impairment [1]. Although the Morris water maze (MWM) can’t reveal a true picture behavioral function of the animal, it was used to measure the hippocampus-dependent spatial memory in AD mice [2,3]. Thus, we also used the Morris water maze to evaluate the spatial memory on our animal models after GBE50 treatments in the study. You give us very important advices and we will use more Behavior Tests to promote our study in the future and reveal a true picture behavioral function of animals.

[1] Shen YX, Xu SY, Wei W, Sun XX, Liu LH, Yang J, Dong C. The protective effects of melatonin from oxidative damage induced by amyloid beta-peptide 25–35 in middle-aged rats. J. Pineal Res. 2002; 32:85–89.
[2] Zheng XY, Lin WJ, Jiang YM, Lu KJ, Wei WJ, Huo QW, Cui SY, Yang XF, Li M, Xu NG, Tang CZ, Song JX. Electroacupuncture ameliorates beta-amyloid pathology and cognitive impairment in Alzheimer disease via a novel mechanism involving activation of TFEB (transcription factor EB). Autophagy. 2021;17(11):3833-3847.
[3] Lin WJ, Li Z, Liang GF, Zhou RJ, Zheng XY, Tao RR, Huo QW, Su CF, Li M, Xu NG, Tang CZ, Song JX. TNEA therapy promotes the autophagic degradation of NLRP3 infammasome in a transgenic mouse model of Alzheimer’s disease via TFEB/TFE3 activation. J Neuroinflammation. 2023;20(1):21.

**Results**

Line 172 – 174 – the statistical analysis in figure 2b and footnote contradicts the arguments/explanation of the results in the result section (line 172-174(. Authors should check their statistical analysis to corroborate this explanation. Similarly in Line 180 – 183 – MAO activity was weaker in CMC and VE, but the comparism cannot be seen in Figure 2D. The statistical analysis does not reveal this effect in Figure 2D. Authors should check the statistical analysis in all the tables and comparisms of the groups and revise appropriately. Line 185 – each them should be corrected. Line 183 – 186 and Figure 2c and e are confusing, diminishing of the enzyme but Authors should revise the issues around the interpretation of the statistical analysis. Group comparism should be done. The report of the results is not specific.

**Author response:** Thank you, the authors have done the editing the statistical analysis in all tables and comparisons of the groups, and the description of each part in the text.

Line 241 – authors should clarify the phrase “caused by aging Aβ.” The methods did not reveal anything related to aging being induced in the animals.

**Author response:** Thank you, the phrase “cause by aging Aβ” is inaccurate, that should be described as “caused by aggregated Aβ_25-35_”. We have done the editing in the text.

Line 265 – 266 - It had been confirmed that Aβ can change the activity of H_2_O_2_-related enzymes in cells in vitro – who confirmed this.

Line 270 – 271 should be revised.

**Author response:** Thank you, the text of Line 265-266 has been edited, that should be “It had been confirmed that the continuous lateral ventricle injection of Aβ_1-40_ can enhance activities of Cu, Zn-SOD in the cytoplasm of rat neocortex, increase activities of MAO-B and reduce activities of CAT and GSH-Px in mitochondria significantly [1].”

The text of Line 270-271 has been revised properly.

[1] Kaminsky YG, Kosenko EA. Effects of amyloid-beta peptides on hydrogen peroxide-metabolizing enzymes in rat brain in vivo. Free Radic Res. 2008;42(6):564-73.

Line 357 – 358 is confusing - include adjusting the ROS metabolism enzyme activity to reduce H_2_O_2_ generation in the mitochondria.

There are lots of grammatical errors in the manuscript. All sections should be checked for proper use of English.

**Author response:** Thank you, the text of Line 357-358 has been edited to be read exactly. The authors have checked all sections and done the editing the grammatical errors in the manuscript.

**Reviewer’s Comments to the Authors:**

**Reviewer #1:**

**Comment 1**

*Ginkgo biloba* capital letter for Ginkgo and italic

ad libitum italic

in vitro italic

in vivo italic

**Author response:** Thank you, the authors have checked all sections of the manuscript and have done edited normatively.

**Comment 2**

Reference for GBE, Aβ_25-35_, Vitamin E, and CMC doses.

**Author response:**

1. According to some studies about GBE50 purchased from the same company, the rats were treated by different dose of GBE50 (20, 40, or 60 mg/kg body weight) by oral gavage for 15days in ischemia reperfusion [1], and the mice were given gavage of 25 mg/kg, 50 mg/kg and 100 mg/kg GBE50 every day from 3 to 15 weeks in high-fat diet [2]. We chose two doses of GBE50 (75 mg/kg and 150 mg/kg) that used usually in our research team and treated rats every day by oral gavage for 15 days after hippocampal injection of Aβ_25–35_ in our study.

2. The dosage of Aβ_25-35_ (20 μg) injected into the rat bilateral hippocampus was obtained from previous study [3].

3. VE was taken at a dose of 100 mg/kg/d used by previous study [4].

4. The 1% sodium carboxymethycellulose (CMC) was used as a solvent for GBE50 [1], as well as for vitamin E. The CMC group was the solvent control to the GBE and VE groups.

[1] Lu SP, Guo X, Zhao PT. Effect of Ginkgo biloba extract 50 on immunity and antioxidant enzyme activities in ischemia reperfusion rats. Molecules. 2011;16(11):9194-206.
[2] Li L, Yang L, Yang F, Zhao XL, Xue SJ, Gong FH. Ginkgo biloba Extract 50 (GBE50) Ameliorates Insulin Resistance, Hepatic Steatosis and Liver Injury in High Fat Diet-Fed Mice. J Inflamm Res. 2021;14:1959-1971.
[3] Shen YX, Xu SY, Wei W, Sun XX, Liu LH, Yang J, et al. The protective effects of melatonin from oxidative damage induced by amyloid beta-peptide 25–35 in middle-aged rats. J. Pineal Res. 2002; 32:85–89.
[4] Alhusaini AM, Alhumaidan SA, Alharbi GM, Alzahrani EA, Sarawi WS, Alomar HA, et al. Cross-Regulation between Autophagy and Apoptosis Induced by Vitamin E and Lactobacillus Plantarum through Beclin-1 Network. Int J Mol Sci. 2022;23(23):15305.

**Comment 3**

Writing the references does not match the Plos One journal method.

**Author response:** Thank you, the authors have checked and formatted all references referred to as the “Vancouver” style to match the Plos One journal.

**Reviewer #2:**

**Comment 1**

It is necessary to investigate the proteins of the internal pathway of apoptosis. The following proteins should be measured.

caspase 3, cleaved caspase 3, caspase 9, cleaved caspase 9

**Author response:** Thanks a lot for your great advices. In this study, we chose to check several proteins (cytochrome c, caspase 3 and CAD) of intraneuronal events following Aβ influence that described by the previous study [1], and the caspase-3 may participate directly in DNA fragmentation, or indirectly activate CAD and cleave the chromatin to result in apoptosis [2]. So, we measured the caspase 3 and CAD proteins to investigate the DNA fragmentation. In the following studies, we want to check the above proteins that you mentioned to promote our research jobs.

[1] Kaminsky YG, Marlatt MW, Smith MA, Kosenko EA. Subcellular and metabolic examination of amyloid-beta peptides in Alzheimer disease pathogenesis: evidence for Abeta(25-35). Exp Neurol. 2010;221(1):26-37.
[2] Klementiev B, Novikova T, Novitskaya V, Walmod PS, Dmytriyeva O, Pakkenberg B, et al. A neural cell adhesion molecule-derived peptide reduces neuropathological signs and cognitive impairment induced by Abeta25-35. Neuroscience. 2007;145(1):209-24.

**Comment 2**

Measurement of amyloid beta peptide is necessary.

**Author response:** Some early research had used as AD-like dementia model by the intracerebroventricular and hippocampal injection of Aβ_25–35_, and the animals shew the cognitive impairment and oxidative damage on few days after the injection of Aβ_25–35_ [1-3]. In this study, we had confirmed the injection of Aβ into the correct point of hippocampus CA1 region by histological detection of the brain (the figures below), and the antioxidant effects of GBE50 administering after injection were evaluated, but the Aβ in the brain was not measured again.

[1] Alkam T, Nitta A, Mizoguchi H, Itoh A, Nabeshima T. A natural scavenger of peroxynitrites, rosmarinic acid, protects against impairment of memory induced by Abeta(25-35). Behav Brain Res. 2007;180(2):139-45.

[2] Klementiev B, Novikova T, Novitskaya V, Walmod PS, Dmytriyeva O, Pakkenberg B, et al. A neural cell adhesion molecule-derived peptide reduces neuropathological signs and cognitive impairment induced by Abeta25-35. Neuroscience. 2007;145(1):209-24.

[3] Shen YX, Xu SY, Wei W, Sun XX, Liu LH, Yang J, Dong C. The protective effects of melatonin from oxidative damage induced by amyloid beta-peptide 25–35 in middle-aged rats. J. Pineal Res. 2002; 32:85–89.

**Comment 3**

The quality of the images is low.

**Author response:** Thank you, we knew the quality of 6^th^ figure was low, which was because of our poor photo technique or low quality of the old fluorescence microscope, but the authors had tried the best to make the images with quality and match to the requirements of PLOS ONE journal.

**Reviewer #3:**

**Comment 1**

In the introduction section, the authors need to mention the impact of mitochondria oxidative stress in the pathogenesis and progression of Alzheimer’s disease.

**Author response:** Thank you, the authors have edited the introduction section according to your suggestions.

**Comment 2**

In the methodology section, the subheading “Determination of H_2_O_2_ and enzyme activities” should be changed to “determination of oxidative stress biomarkers and enzymes activities”.

**Author response:** Thanks a lot, the authors have edited this text in the manuscript.

**Comment 3**

In the discussion section, authors need to thoroughly discuss the impact of their findings on the role of mitochondrial oxidative stress in Alzheimer’s disease. The discussion should provide a link of all the results for easy understanding on the therapeutic effect of *Ginkgo biloba* extract on Alzheimer’s disease.

**Author response:** Thank you, the authors have edited the discussion section properly, and provide a conclusion of all results for easy understanding on the effect of GBE in AD-like rats.

**Comment 4**

What are the bioactive components of the extract? It is imperative that the authors identify the bioactive compounds of the extracts which may be responsible for its therapeutic effect.

**Author response:** The main ingredients of standardized *Gingko biloba* extract are flavonoids

and terpenoids [1]. There are some differences in the antioxidant capacity of the four typical components of *Ginkgo biloba* extract including ginkgo flavone (GF), ginkgolide (G), procyanidins (OPC), and organic acids (OA), and any two members of them can exhibit apparent synergistic effects [2]. In our research team, we are going to identify the bioactive compounds of GBE which may be responsible for its therapeutic effect. Thank you for great advices.

[1] Xie LM, Zhu Q, Lu JH. Can we use *Ginkgo biloba* extract to treat Alzheimer's disease? Lessons from preclinical and clinical studies. Cells. 2022;11(3):479.

[2] Zhang LH, Zhu CY, Liu XQ, Su EZ, Cao FL, Zhao LG. Study on Synergistic Antioxidant Effect of Typical Functional Components of Hydroethanolic Leaf Extract from *Ginkgo Biloba* In Vitro. Molecules. 2022;27(2):439.
