# Supplementary material for: Ginkgo biloba extract inhibits hippocampal neuronal injury caused by mitochondrial oxidative stress in a rat model of Alzheimer’s disease
Source: PLoS One. 2024 Aug 6;19(8):e0307735. doi: 10.1371/journal.pone.0307735 (PMC11302898; doi:10.1371/journal.pone.0307735)
Supplement: Attachment — Submitted filename: Response to Reviewers.docx [file pone.0307735.s004.docx]

**Journal: PLOS NOE**

**Title:** *Ginkgo biloba* extract inhibits hippocampal neuronal injury caused by mitochondrial oxidative stress in Alzheimer's disease rat

**PONE-D-23-22230R1**

**Response to Reviewers**

**Dear Dr. Olasehinde and reviewers,**

First I would like to thank you for giving us the opportunity to submit a revised draft of the manuscript “*Ginkgo biloba* extract inhibits hippocampal neuronal injury caused by mitochondrial oxidative stress in Alzheimer's disease rat” for publication in the Journal of PLOS ONE. We sincerely appreciate the time and effort that you and three reviewers dedicated to provide the valuable comments on our manuscript. All the insightful comments were considered carefully and the manuscript was revised with tracked changes as necessary based on the suggestions made by reviewers. Attached please find the point-by-point response to the reviewers’ comments and concerns. Should you have any questions or concerns, please contact me.

Sincerely,

Chenyi Xia

**Editor’s Comments to the Authors:**

The authors are yet to provide the information on the characterization and identification of the bioactive constituents of GBE. The journal policy requires that information be provided on identification and characterization of extracts.

**Author response:** Thanks a lot for your great advices. The compounds of GBE50 had been identified by UPLC-Q/TOF-MS analysis in previous study [1], and this drug was from the same company as the one we bought. We also upload a supporting information of the certificate of analysis for GBE50 provided by the company.

[1] Ke J, Li MT, Huo YJ, Cheng YQ, Guo SF, Wu Y, et al. The Synergistic Effect of Ginkgo biloba Extract 50 and Aspirin Against Platelet Aggregation. Drug Des Dev Ther. 2021;15:3543-60.
