# Supplementary material for: Ginkgo biloba extract inhibits hippocampal neuronal injury caused by mitochondrial oxidative stress in a rat model of Alzheimer’s disease
Source: PLoS One. 2024 Aug 6;19(8):e0307735. doi: 10.1371/journal.pone.0307735 (PMC11302898; doi:10.1371/journal.pone.0307735)
Supplement: Attachment — Submitted filename: Response to Reviewers.docx [file pone.0307735.s005.docx]

**Journal: PLOS NOE**

**Title:** *Ginkgo biloba* extract inhibits hippocampal neuronal injury caused by mitochondrial oxidative stress in a rat model of Alzheimer's disease

**PONE-D-23-22230R2**

**Response to Reviewers**

**Dear Dr. Olasehinde and reviewers,**

First I would like to thank you for giving us the opportunity to submit a revised draft of the manuscript “*Ginkgo biloba* extract inhibits hippocampal neuronal injury caused by mitochondrial oxidative stress in a rat model of Alzheimer's disease” for publication in the Journal of PLOS ONE. We sincerely appreciate the time and effort that you and three reviewers dedicated to provide the valuable comments on our manuscript. All the insightful comments were considered carefully and the manuscript was revised thoroughly with tracked changes as necessary based on editor’s suggestions. Attached please find the point-by-point response to the editor’s comments and concerns. Besides, we edited three figures properly to match with the results. Should you have any questions or concerns, please contact me.

Sincerely,

Chenyi Xia

**Editor’s Comments to the Authors:**

Introduction - Line 61 - EGb761, as a commercial product of GBE, - what is the difference between EGb761 and GBE. Does processing of the commercial product affect the levels of the bioactive constituents.

**Author response:** Thanks a lot for your great advices. GBE50, a standardized product of *Ginkgo biloba* extract (GBE) that is the equivalent to the standardized German product as EGb761, has been approved for clinically use by the China Food and Drug Administration (approval number: Z20000049) [1]. GBE50 contains *Ginkgo biloba* flavonoids (44%) and terpene lactones (6%), EGb761 includes *Ginkgo biloba* flavonoids (22-27%) and terpene lactones (5-7%) which is mentioned in Introduction of the manuscript. Distinguishing technique in production process contributes to different levels of the bioactive constituents in GBE50 and EGb761, but how the processing of the commercial product affects the difference is commercial secret we don’t know. A recent study has stated that the biological activities of GBE produced by different production processes are different [2]. In addition, we have uploaded a supporting information of the certificate of analysis for GBE50 provided by the company.

[1] Ke J, Li MT, Huo YJ, Cheng YQ, Guo SF, Wu Y, et al. The Synergistic Effect of *Ginkgo biloba* Extract 50 and Aspirin Against Platelet Aggregation. Drug Des Dev Ther. 2021;15:3543-60.

[2] Zhang L, Zhu C, Liu X, Su E, Cao F, Zhao L. Study on Synergistic Antioxidant Effect of Typical Functional Components of Hydroethanolic Leaf Extract from *Ginkgo Biloba* In Vitro. Molecules. 2022; 27(2):439. doi: 10.3390/molecules27020439.

Introduction - Line 64 and 65 – what does authors mean and its effect component contains……this is not appropriate and should be revised.

**Author response:** Thank you. The description of “its effect components contains……” has been edited properly. These words express that the bioactive components have different levels in EGb761 and GBE50. GBE50 contains *Ginkgo biloba* flavonoids (44%) and terpene lactones (6%), but EGb761 includes *Ginkgo biloba* flavonoids (22-27%) and terpene lactones (5-7%) which is mentioned in Introduction of the manuscript. They are two different commercial product of *Ginkgo biloba* extract.

Introduction - Line 65 – authors only mentioned the class of compounds – flavonoids and lactones present in GBE-50. What are the specific constituents present in GBE50 which has been published as indicated in reference [21]. This should be stated appropriately

**Author response:** Thank you. The specific constituents present in GBE50 has been stated in Materials of the manuscript. GBE50 with a composition of ≥ 44% Ginkgo biloba total flavonoids (≥ 24% flavonol glycosides, ≥ 20% free flavones), ≥ 6% lactones (≥ 3.1% Ginkgo biloba lactones and ≥ 2.9% bilobalide) and ≤ 5 ppm ginkgolic acids has been identified via UPLC-Q/TOF-MS analysis.

Results - No sequential order for the presentation of the results. Presentation of results from Figure 2D must come be after 2B and 2C.

**Author response:** Thank you very much. The presentation of results has been adjusted appropriately.

- Authors should note that the constituents of GBE50 majorly contributed either individually or synergistically to the activities reported in this study. The role of the constituents in the biochemical mechanism of GBE is missing in the discussion. Furthermore, the antioxidant activity of GBE50 which has been reported may also play an important role in the observed activity reported, but all these arguments are missing.

**Author response:** Thanks for the comments. The arguments about antioxidant effects of essential constituents of GBE and antioxidant activity of GBE50 have been edited in the discussion of manuscript.

- The manuscript requires extensive English editing

**Author response:** Thanks a lot. The manuscript has been edited by a professional native speaker. The detail please refer to the manuscript (with track changes in word).

In addition, we edited three figures (Fig 3, Fig 4 and Fig5) into a new figure (Fig 3 in the manuscript) properly to match with the results. Fig 1 and Fig 2 were kept the original, and Fig 6 of the previous edition of manuscript was switch to Fig 4 in the current manuscript.
