# Supplementary material for: Ginkgo biloba extract inhibits hippocampal neuronal injury caused by mitochondrial oxidative stress in a rat model of Alzheimer’s disease
Source: PLoS One. 2024 Aug 6;19(8):e0307735. doi: 10.1371/journal.pone.0307735 (PMC11302898; doi:10.1371/journal.pone.0307735)
Supplement: Attachment — Submitted filename: Response to Reviewers.docx [file pone.0307735.s006.docx]

**Journal: PLOS NOE**

**Title:** *Ginkgo biloba* extract inhibits hippocampal neuronal injury caused by mitochondrial oxidative stress in a rat model of Alzheimer's disease

**PONE-D-23-22230R3**

**Response to Reviewers**

**Dear Dr. Olasehinde and reviewers,**

First I would like to thank you for giving us the opportunity to submit a revised draft of the manuscript “*Ginkgo biloba* extract inhibits hippocampal neuronal injury caused by mitochondrial oxidative stress in a rat model of Alzheimer's disease” for publication in the Journal of PLOS ONE. We sincerely appreciate the time and effort that you and three reviewers dedicated to provide the valuable comments on our manuscript. All the insightful comments were considered carefully and the manuscript was revised thoroughly with tracked changes as necessary based on editor’s suggestions. Attached please find the point-by-point response to the editor’s comments and concerns. Briefly, we edited a part of Discussion appropriately and cited an original research article in which GBE50 was sufficiently characterized, so the Reference list was adjusted properly. Should you have any questions or concerns, please contact me.

Sincerely,

Chenyi Xia

**Editor’s Comments to the Authors:**

Authors have significantly improved the manuscript. However, authors still need to sufficiently present or justify that GBE50 has been sufficiently characterized as per journal requirement. Authors cited a review article (reference 58) to indicate the constituents of GBE50. Authors should rather use an original research article that has previously characterized GBE50.

**Author response:** Thanks a lot. We checked the original research article that has characterized GBE50 from 2014 to the present on PubMed, and found only two such papers.

In the first article published in 2021 [1] that we cited it as Reference 21 in our manuscript, the compounds in GBE50 were identified by UPLC-Q/TOF-MS analysis. 73 compounds were identified, including terpene lactones, flavonoids and their glycosides, organic acids, flavanols and biflavones. Among them, 39 components were compared with reference substances and the relevant information was searched in databases such as PubChem and TCMSP. The related information for each component for shown in Table 1 of this article.

[1] Ke J, Li MT, Huo YJ, Cheng YQ, Guo SF, Wu Y, et al. The Synergistic Effect of *Ginkgo biloba* Extract 50 and Aspirin Against Platelet Aggregation. Drug Des Dev Ther. 2021;15:3543-60. doi: 10.2147/Dddt.S318515. PMID: 34429584.

In the second article published in 2023 [2], the constituents in GBE50 were analyzed by UHPLC-Q-Exactive Orbitrap HRMS. As illustrated in Table S1 of this article, altogether 38 compounds were analyzed in GBE50, including 23 flavonoids, 5 biflavonoids, 4 catechins, 5 terpene lactones, and 1 organic acid.

[2] Zhang YN, Zhu GH, Liu W, Xiong Y, Hu Q, Zhuang XY, et al. Discovery and characterization of the covalent SARS-CoV-2 3CL^pro^ inhibitors from *Ginkgo biloba* extract *via* integrating chemoproteomic and biochemical approaches. Phytomedicine. 2023;114:154796. doi: 10.1016/j.phymed.2023.154796. PMID:37037086.

**Table S1** Identification of chemical constituents of *Ginkgo biloba* extract 50*.*

| No. | Rt  (min) | Ion mode | Measured mass/Da | Calculated mass/Da | Error/ppm | Molecular formula | Identification |
| --- | --- | --- | --- | --- | --- | --- | --- |
|  |  |  |  |  |  |  |  |
| 1* | 0.97 | [M-H]^-^ | 191.05510 | 191.05501 | 0.447 | C_7_H_12_O_6_ | Quinic Acid |
| 2* | 4.25 | [M-H]^-^ | 153.01794 | 153.01824 | -1.929 | C_7_H_6_O_4_ | Protocatechuic Acid |
| 3* | 8.33 | [M-H]^-^ | 305.06647 | 305.06558 | 2.92 | C_15_H_14_O_7_ | Gallocatechin |
| 4* | 8.5 | [M-H]^-^ | 289.07159 | 289.07066 | 3.201 | C_15_H_14_O_6_ | Catechin |
| 5* | 10.93 | [M-H]^-^ | 325.09274 | 325.09179 | 2.91 | C_15_H_18_O_8_ | Bilobalide |
| 6* | 12.05 | [M-H]^-^ | 289.07166 | 289.07066 | 3.443 | C_15_H_14_O_6_ | Epicatechin |
| 7 | 14.48 | [M-H]^-^ | 423.12964 | 423.12857 | 2.521 | C_20_H_24_O_10_ | Ginkgolide J |
| 8* | 15.14 | [M-H]^-^ | 439.12433 | 439.12349 | 1.918 | C_20_H_24_O_11_ | Ginkgolide C |
| 9* | 16.31 | [M-H]^-^ | 625.14081 | 625.13993 | 1.415 | C_27_H_30_O_17_ | Myricetin-3-*O*-[α-L-rhamnopyranosyl-(1→6)-β-D-glucopyranoside] |
| 10* | 16.91 | [M-H]^-^ | 755.20416 | 755.20292 | 1.642 | C_33_H_40_O_20_ | Manghaslin |
| 11* | 17.86 | [M-H]^-^ | 287.05594 | 287.05501 | 3.224 | C_15_H_12_O_6_ | Aromadendrin |
| 12* | 18.51 | [M-H]^-^ | 407.13452 | 407.13366 | 2.116 | C_20_H_24_O_9_ | Ginkgolide A |
| 13* | 18.64 | [M-H]^-^ | 739.20966 | 739.20801 | 2.239 | C_33_H_40_O_19_ | Clitorin |
| 14* | 18.87 | [M-H]^-^ | 463.0882 | 463.08710 | 2.37 | C_21_H_20_O_12_ | Isoquercitrin |
| 15* | 18.99 | [M-H]^-^ | 609.14612 | 609.14501 | 1.82 | C_27_H_30_O_16_ | Rutin |
| 16* | 18.99 | [M-H]^-^ | 769.21991 | 769.21857 | 1.742 | C_34_H_42_O_20_ | Typhaneoside |
| 17* | 19.12 | [M-H]^-^ | 423.12979 | 423.12857 | 2.876 | C_20_H_24_O_10_ | Ginkgolide B |
| 18* | 19.29 | [M-H]^-^ | 623.16187 | 623.16066 | 1.94 | C_28_H_32_O_16_ | Isorhamnetin-3-*O*-Neohespeidoside |
| 19* | 19.85 | [M-H]^-^ | 639.1568 | 639.15558 | 1.915 | C_28_H_32_O_17_ | Laricitrin 3-*O*-Rutinoside |
| 20* | 20.91 | [M-H]^-^ | 609.14587 | 609.14501 | 1.41 | C_27_H_30_O_16_ | Quercetin-3-*O*-D-Glucopyranosyl-(1-2)-L-Rhamnoside |
| 21* | 21.25 | [M-H]^-^ | 447.09311 | 447.09219 | 2.063 | C_21_H_20_O_11_ | Astragalin |
| 22* | 21.39 | [M-H]^-^ | 593.15106 | 593.15010 | 1.624 | C_27_H_30_O_15_ | Nicotiflorin |
| 23* | 21.79 | [M-H]^-^ | 477.10355 | 477.10275 | 1.672 | C_22_H_22_O_12_ | Isorhamnetin-3-*O*-Glucoside |
| 24* | 22 | [M-H]^-^ | 623.16162 | 623.16066 | 1.539 | C_28_H_32_O_16_ | Narcissoside |
| 25* | 22.43 | [M-H]^-^ | 653.17267 | 653.17123 | 2.211 | C_29_H_34_O_17_ | Syringetin-3-*O*-Rutinoside |
| 26* | 23.21 | [M-H]^-^ | 593.151 | 593.15010 | 1.523 | C_27_H_30_O_15_ | Kaempferol 3-*O*-β-D-Glucopyranosyl-(1→2)-α-L-Rhamnop  yranoside |
| 27* | 23.02 | [M-H]^-^ | 755.18292 | 755.18179 | 1.496 | C_36_H_36_O_18_ | Quercetin-3-*O*-(6'''-Trans-*p*-Coumaroyl-2''-Glucosyl)Rhamnoside |
| 28* | 24.12 | [M-H]^-^ | 301.03516 | 301.03428 | 2.926 | C_15_H_10_O_7_ | Quercetin |
| 29* | 25.04 | [M-H]^-^ | 739.18823 | 739.18688 | 1.832 | C_36_H_36_O_17_ | Kaempferol-3-*O*-(6'''-Trans-*p*-Coumaroyl-2''-Glucosyl)Rhamnoside |
| 30* | 25.25 | [M-H]^-^ | 285.04041 | 285.03936 | 3.668 | C_15_H_10_O_6_ | Luteolin |
| 31* | 27.27 | [M-H]^-^ | 285.04028 | 285.03936 | 3.212 | C_15_H_10_O_6_ | Kaempferol |
| 32* | 27.73 | [M-H]^-^ | 269.04547 | 269.04445 | 3.792 | C_15_H_10_O_5_ | Apigenin |
| 33* | 28.14 | [M-H]^-^ | 315.05106 | 315.04993 | 3.589 | C_16_H_12_O_7_ | Isorhamnetin |
| 34* | 33.41 | [M-H]^-^ | 537.08246 | 537.08162 | 1.558 | C_30_H_18_O_10_ | Amentoflavone |
| 35* | 35.25 | [M-H]^-^ | 551.09808 | 551.09727 | 1.464 | C_31_H_20_O_10_ | Bilobetin |
| 36* | 39.65 | [M-H]^-^ | 565.11383 | 565.11292 | 1.605 | C_32_H_22_O_10_ | Ginkgetin |
| 37* | 41.01 | [M-H]^-^ | 565.11401 | 565.11292 | 1.923 | C_32_H_22_O_10_ | Isoginkgetin |
| 38* | 41.2 | [M-H]^-^ | 579.12982 | 579.12857 | 2.153 | C_33_H_24_O_10_ | Sciadopitysin |

* The labeled compounds were confirmed by using the reference standard compounds.

Since both articles are original research articles in which GBE50 was sufficiently characterized to indicate the class of compounds and individual components present, and the first article was cited as Reference 21 in our manuscript, we edited a part of our discussion properly and cited the second article as a new Reference 58. The detail please refer to the manuscript (with track changes in word).

**Journal Requirements to the Authors:**

Please review your reference list to ensure that it is complete and correct. If you have cited papers that have been retracted, please include the rationale for doing so in the manuscript text, or remove these references and replace them with relevant current references. Any changes to the reference list should be mentioned in the rebuttal letter that accompanies your revised manuscript. If you need to cite a retracted article, indicate the article’s retracted status in the References list and also include a citation and full reference for the retraction notice.

**Author response:** Thank you. We double-checked our reference list to ensure that it was complete and correct, the information of Reference 2 and 7 were supplemented sufficiently, and no cited papers to date was retracted. Because a new article was citied as Reference 58, the list of Reference followed it had to be adjusted properly and did it.
